# Improving the sensitivity of the hop index in patients with an ACL deficient knee by transforming the hop distance scores

**DOI:** 10.1186/1471-2474-7-9

**Published:** 2006-02-01

**Authors:** Siobhan O'Donnell, Scott G Thomas, Paul Marks

**Affiliations:** 1 Centre for Studies of Physical Function, Orthopaedic & Arthritic Institute, Sunnybrook and Women's College Health Science Centre, Toronto, Ontario, Canada; 2 Graduate Department of Rehabilitation Science, University of Toronto, Toronto, Ontario, Canada; 3 Department of Orthopaedic Surgery, Orthopaedic & Arthritic Institute, Sunnybrook and Women's College Health Science Centre Toronto, Ontario, Canada; 4 Department of Surgery, Faculty of Medicine, University of Toronto, Toronto, Ontario, Canada

## Abstract

**Background:**

The one leg hop for distance is one of the most commonly employed functional tests utilized in the evaluation of the ACL deficient and reconstructed patient. While the reliability of the hop test scores has been well established, validity studies have revealed low sensitivity rates in detecting functional limitations using the hop index (the ratio or percentage of limb performance). However, the impact of the inherent limitations associated with the hop index have not been investigated to date. One specific limitation relates to the impact of the differences in the underlying hop distance scores. Therefore, this pilot study set out to determine: 1) the impact that between limb differences in hop distance has on the sensitivity of the hop index in detecting functional limitations and; 2) whether a logarithmic transformation of the underlying hop distance scores improves the sensitivity of the hop index.

**Methods:**

A cross sectional design involving the evaluation of one leg hop for distance performance in a consecutive sample of 10 ACL deficient males with an isolated ACL tear awaiting reconstructive surgery and nine gender, age-matched controls.

**Results:**

In the ACL deficient, the hop index was associated with the distance hopped on the non-injured limb (r = -0.66, p = 0.04) but not on the injured limb. Transformation (logarithmic) of the hop distance scores and re-calculation of the hop index using the transformed scores increased the sensitivity of the hop index in the detection of functional limitations from 20 to 60% and 50 to 70% using the normal limb symmetry reference norms of ≥ 85% and 90% respectively.

**Conclusion:**

The distance hopped on the non-injured limb is a critical factor in detecting functional limitations using the hop index in patients with an ACL deficient knee. Logarithmic transformation of the hop distance scores minimizes the effect of the arithmetic differences between limbs however; the sensitivity of the hop index in detecting abnormal limb symmetry remains low.

## Background

The one-leg hop for distance (OLHD) has become one of the most commonly employed functional tests utilized in the evaluation of the ACL deficient (ACLD) or reconstructed patient, especially since its inclusion in the International Knee Documentation Committee (IKDC) standard knee ligament evaluation [1-10]. It is considered a relevant task to assess since the functional limitations in the ACLD are most evident during the performance of such athletic activities as a result of the capsuloligamentous sagittal load on landing [11-14]. Furthermore, it can be conducted in any clinical setting and requires minimal staff training, time and equipment [15-19].

The OLHD scores include the hop distance and hop index, which is the ratio or percentage of hop distance achieved on one limb relative to the other. Hop index reference norms are used widely as clinical benchmarks for establishing normal versus abnormal OLHD performance. A normal hop index has been shown to be ≥ 85% by Barber et al [16] and ≥ 90% by Daniel et al [9,20]. These reference norms were empirically established by noting > 90% of subjects without a history of ACL injury had a hop index of ≥ 85% [16] and ≥ 90% [9,20], both of which are cited in the literature. Therefore, to allow for a comparison of our results with other studies, both reference norms are referred to throughout this report.

While the reliability of the OLHD has been well established in terms of the consistency of patient scores[17,18,21,22], previous validity studies have revealed low sensitivity rates in detecting abnormal limb symmetry [16,19] in the ACLD population. However, limitations associated with the hop index have not been dealt with appropriately. One specific limitation relates to the impact of the differences in the underlying hop distance scores.

Distance hopped on the non-injured leg may vary widely according to natural differences in athletic ability and muscle strength however; variation in the distance hopped on the ACL injured leg is likely constrained due to impairment. Consequently, the sensitivity of the hop index in detecting functional limitations in the ACLD would increase incrementally with the distance hopped on the non-injured leg. A logarithmic transformation of the hop distance scores could improve the sensitivity of the hop index by making it more sensitive to the proportional variation in the scores relative to arithmetic differences for those ACLD patients who do not hop as far on the non-injured limb.

In view of the above, the objectives of this pilot study were twofold:

1. To assess the impact that between limb differences in hop distance has on the sensitivity of the hop index in detecting functional limitations in the ACLD patient requiring reconstructive surgery and;

2. To assess whether a logarithmic transformation of the hop distance scores increases the sensitivity of the hop index within this population.

## Methods

### Subjects

Ten consecutive males on a waiting list for ACL reconstructive surgery were recruited. The inclusion of the ACL deficient patient was dependent upon them having a grade 2 or 3 ACL ligamentous laxity on manual testing, being at least 6 weeks post injury and the absence of other ligamentous involvement. Ten gender and age-matched healthy Controls were recruited from the community through the use of flyers and word-of-mouth.

The ACLD patients were tested 5.3 to 60 months post injury and were similar in mean (+/- SEM) age in comparison to Controls (28.4 (+/- 2.74) and 28.4 (+/- 1.11) years respectively). All ACLD patients demonstrated normal extension range of motion on the injured limb (less than 3-degree difference between limbs), seven had normal flexion (between 0 to 5-degree difference between limbs) and three had nearly normal flexion (between 6 to 15 degree difference between limbs). None of the ACLD patients had evidence of swelling at the time of testing.

Self-report activity levels were evaluated using the Tegner Activity Scale (score range from zero to ten, with zero representing no activity secondary to the knee condition). The ACLD patients engaged in significantly less demanding work/sport related activities after their ACL injury (median (25th; 75th percentiles) pre-injury: 7.5 (7;9) and post-injury 4.5 (4;6); p = 0.004). However, the difference between the ACLD patients and Controls at the time of testing was not significantly different (4.5 (4;6) versus 6.5 (4;8) respectively; p = 0.082).

### OLHD

OLHD performance was evaluated with the subject's hands behind their back, to minimize the effect of arm swing and subjects were instructed to stand on one limb and hop as far forward as possible. Placement of the other foot upon landing was not permitted however; subjects were not required to hold their landing position. A total of three hop trials on each limb were performed. Practice trials were not permitted and the order of limb testing was randomly determined. Subjects warmed up for five minutes on a stationary bike prior to testing.

The hop distance was calculated by measuring the distance traveled from heel to heel from the beginning to final standing position. The longest distance of the three trials for each leg (as opposed to the average) was used to calculate the hop index for ease in computing as it has been shown that the different analysis strategies have no effect on the hop index [18]. Leg dominance was determined by asking the subject to identify the leg with which they would preferably kick a ball.

### Informed consent

Informed consent was obtained prior to testing, subjects were tested on one occasion only and all testing was performed by the same physical therapist. The research ethics committee of the hospital and its affiliated academic institution granted approval of the study protocol.

### Data analysis plan

The validity of the OLHD in depicting functional limitations in an ACLD knee was determined through an interpretation of the diagnostic indices (i.e. sensitivity and specificity whereby the closer they are to 100%, the more sensitive and specific the test). The impact of limb differences in distance hopped on the hop index was explored by analyzing scatterplots of the hop distance scores, examining the association between limb hop distance scores and investigating the relationships between the hop distance scores and hop index. In order to minimize the arithmetic variation between limb performance of the ACLD and Controls, the hop distance scores were transformed using a logarithmic (log_10_) transformation, although logarithms to any base would have had the same effect [23-25]. Following transformation of the hop distance scores, the hop index was re-calculated and the diagnostic test rates were re-analyzed.

### Statistical analysis

Statistical computations were performed using SigmaStat (Version 2.03, SPSS Inc.). For comparisons involving parametric data, independent and paired t-tests were employed and for non-parametric data, the Mann-Whitney Rank Sum and Wilcoxon-Signed Rank tests were used. Linear regression analyses were conducted using the Pearson Product Correlation Coefficient. All differences were deemed significant at the p < 0.05 level.

## Results

### Hop distance scores

Within group comparisons revealed a significant difference in distance hopped between limbs within the ACLD group (mean difference = 20 cm; p = 0.006) but not within the Controls (5 cm; p = 0.21). Also, between group comparisons demonstrated a significant difference in the distance hopped on the ACLD limb relative to the average limb performance (dominant + non-dominant / 2) of the Controls (19 cm; p = 0.013) but not between the non-injured limb of the ACLD patients and Controls (3 cm; p = 0.67).

### Hop index scores (original) relative to reference norms

The percentage of ACLD patients with an abnormal hop index using the hop index reference norm ≥ 85% was 20% and ≥ 90%, 50%. Whereas, 100% of the Controls demonstrated a normal hop index irrespective of the reference norm employed (Table 1).

### The impact of limb differences in distance hopped on the sensitivity of the hop index

**Figure 1:**
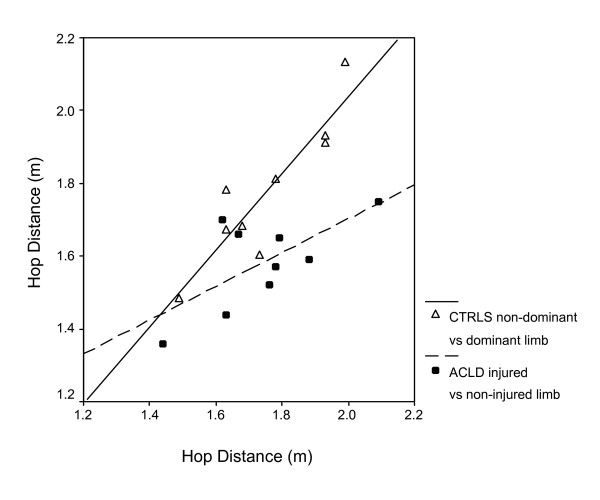
Pearson Product Moment Correlation plots illustrating the association in hop distance performance between limbs within the ACLD (r = 0.68; p = 0.04) and Control groups (r = 0.91; p = < 0.001).

Scatterplots of the hop distances achieved revealed less variation in the hop distance scores on the ACLD limb compared to the non-injured limb of the ACLD patients and Controls. Furthermore, a comparison of the relationship between limbs in hop distance performance revealed a weaker association within the ACLD group (r = 0.68; p = 0.04) compared to Controls (r = 0.91; p = <0.001) (Figure 1).

**Figure 2:**
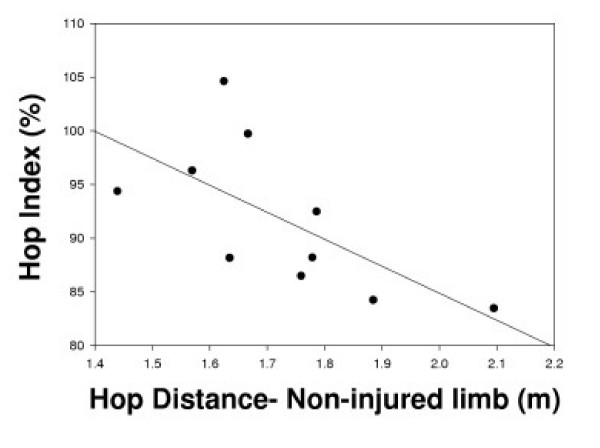
A Pearson Product Moment Correlation plot illustrating the association between the hop index (injured/non-injured limb hop distance × 100%) and the hop distance on the non-injured limb in the ACLD group (r = -0.66, p = 0.04).

In the ACLD group, the distance achieved on the injured limb was not associated with the hop index (r = 0.10; p = 0.79) however, on the non-injured limb, the correlation between the hop index and hop distance was significant (r = -0.66, p = 0.04) (Figure 2). The Controls did not demonstrate an association between the hop distance (average of limbs) and the hop index (r = 0.32; p = 0.40).

### Hop index scores (transformed) relative to reference norms

The percentage of ACLD patients with an abnormal hop index of < 85% increased from 20 to 60% when the hop index scores were recalculated using the transformed hop distance scores. In a like manner, the percentage with an abnormal hop index of < 90%, increased from 50 to 70%. The percentage of Controls who demonstrated a normal hop index basically remained unchanged (Table 2).

## Discussion

The most important finding of this study was that a mathematical transformation of the hop distance scores improved the sensitivity of the hop test index in detecting abnormal limb symmetry in the ACLD patient.

The clustered appearance of the hop distance scores on the injured limb, and the weaker association in hop distance between limbs, led us to believe that the ACLD patients performed fairly similarly on their injured limb regardless of their performance on the non-injured limb. As a result, those ACLD patients that hopped farther on their non-injured limb were more likely to demonstrate an abnormal hop index according to reference norms. Therefore, controlling for differences in the distance hopped on the non-injured limb would ensure that the absolute hop distance was less of a critical factor in the sensitivity of the hop index.

Distributions of clinical measures associated with functional status often contain extraneous and/or insufficient variation associated with outliers or clustering hence, transformation of the associated scores is a commonly utilized strategy to make a measure more useful for its intended purpose [24]. In our study, transforming the hop distance scores enabled a recalculation of the hop index without concern for the differences in the hop distances achieved on the ACLD patient's non-injured limb. In doing so, there was an increase in the percentage of ACLD patients who demonstrated an abnormal hop index using either the reference norm defined by Barber et al. (1990)[16] or Daniel et al. (1982 and 1988)[9,20]. Whereas the percentage of Controls who demonstrated a normal hop index basically remained unchanged.

When a measure is being used for a specific clinical application such as diagnosis, prognosis and/or responsiveness to treatment, the decision regarding the most appropriate transformation should be based on theoretical considerations of how variation in the measure will impact its performance in practice [24]. This will increase the likelihood that results based on this transformation are generalizable to the population of interest rather than an artifact of a given sample. One criterion in assessing how well a data transformation will perform across different samples is to determine whether it can minimize increases in the standard deviation in relation to increases in the mean [24]. Upon examination of our data, the standard deviation increased in proportion to the mean for the original hop distance scores of the ACLD limb versus the non-injured limbs of the ACLD and Controls. While the logarithmic transformation served to equalize the standard deviations among these hop distance scores (Table 3). However, despite the improvement in the sensitivity of the hop index using the transformed scores, the sensitivity of the hop index remains low given that approximately one third of the ACLD patients were classified as having a normal hop index.

Many methods of assessing neuromuscular function have demonstrated that both limbs are affected after a unilateral ACL injury [26-28]. Therefore, it is possible that the patient with an ACL injury has a normal hop index relative to reference norms but the distance hopped on both limbs is shorter when compared with individuals with normal knees. Another potential explanation is that the OLHD is not challenging enough to elicit the functional limitations in those ACLD patients that experience dynamic instability in sporting or other demanding activities.

Given our limited sample size, this pilot study was under powered hence; further validation of this approach is required prior to recommending its use within a clinical setting. Future studies should include ACLD males and females, across the spectrum of injury, with varying levels of chronicity [29]. Also, consideration should be given to employing a Receiver Operator Characteristic Curve in order to select an appropriate cut-off point in the determination of a normal versus abnormal hop index in the ACLD versus non-injured, healthy population [30].

Should the sensitivity of the hop index in detecting functional limitations remain low in future work, consideration should be given to the development of a more challenging lower limb functional test in the assessment of ACL injuries. The test of choice should be objective, reliable and provide an independent assessment of limb performance such that the opposite limb can be used for comparison. Finally, it ought to simulate stresses about the knee encountered during activities relevant to the athlete, stress in multiple planes of motion and incorporate 'reactive', as opposed to planned, testing conditions.

In closing, while performance tests such as the OLHD are very useful to the clinician when attempting to describe a patient's functional status and disability, they should not be used in isolation. Other clinical assessments including knee joint laxity, muscle performance, proprioception and self-report measures of function capture different aspects of physical performance and when used in combination, more comprehensively describe the patient's status at a given point in time [16,31].

## Conclusion

The distance hopped on the non-injured limb is potentially a critical factor in the sensitivity of the hop index in detecting functional limitations in the ACLD. Transforming the hop distance scores, to minimize the effect of the arithmetic differences between limbs, may improve the sensitivity of the hop index in detecting functional limitations in the ACLD patient. Further validation of this approach is required prior to recommending its use within the clinical realm.

## Competing interests

The author(s) declare that they have no competing interests.

## Authors' contributions

SO'D conceived and designed the study, acquired the data, conducted the data analysis and drafted the manuscript. ST contributed to the conception and the design of the study, assisted with the interpretation of the analysis and reviewed the manuscript critically for intellectual content. PM assisted in the recruitment of subjects and contributed to the overall coordination of the study. All authors read and approved the final manuscript.

## Pre-publication history

The pre-publication history for this paper can be accessed here:



## Figures and Tables

**Table 1:** Original hop index scores

	ACLD knees	Normal knees
Abnormal limb symmetry <85% (<90%)	20% (50%)	0% (0%)
Normal limb symmetry ≥85% (≥90%)	80% (50%)	100% (100%)

Sensitivity, specificity and other diagnostic indices of the original hop index scores using ≥85% (≥90%) hop index reference norms.

**Table 2:** Transformed (log_10_) hop index scores:

	ACLD knees	Normal knees
Abnormal limb symmetry <85% (<90%)	60% (70%)	0% (10%)
Normal limb symmetry ≥85% (≥90%)	40% (30%)	100% (90%)

Sensitivity, specificity and other diagnostic indices of the hop index using the transformed hop distance scores using ≥85% (≥90%) hop index reference norms.

**Table 3:** Descriptive statistics for the original and log_10 _transformed hop distance scores

	**Hop distance (m)**	**ACLD**		**CONTROLS**	
		**Injured**	**Non-injured**	**Non-dominant**	**Dominant**
**Original**	mean (SD)	1.58 (0.12)	1.72 (0.18)	1.78 (0.20)	1.75 (0.17)
	SD/mean	0.08	0.10	0.11	0.10
**Transformed**	mean (SD)	0.20 (0.03)	0.23 (0.04)	0.25 (0.05)	0.24 (0.04)
	SD/mean	0.15	0.17	0.20	0.17

Descriptive statistics for the original and log_10 _transformed hop distance scores including the mean, standard deviation (SD) and SD expressed relative to the mean.
